# Editorial: Emerging areas in literature-based discovery

**DOI:** 10.3389/frma.2023.1122547

**Published:** 2023-01-19

**Authors:** Yakub Sebastian, Neil R. Smalheiser

**Affiliations:** ^1^Charles Darwin University, Darwin, NT, Australia; ^2^University of Illinois at Chicago, Chicago, IL, United States

**Keywords:** literature-based discovery, fundamental issues, knowledge gaps, usability, data visualization, network analysis

Literature-Based Discovery (LBD) aims at generating novel and actionable knowledge from vast, diverse, and seemingly disconnected fragments of information. Recent advances in LBD are focused on experimenting with state-of-the-art algorithms such as knowledge graphs, embeddings, deep learning, etc. and expanding the practical use cases of LBD (see for example Sosa and Altman, 2022; Syafiandini et al., 2022; Zhou et al., 2022). Notwithstanding these valuable developments, much can still be done to address more fundamental issues in the field. This Research Topic presents progress in expanding our understanding of diverse gaps in the literature (Peng et al.), improving end-user experiences (Henry et al.), creating a scalable and visual-driven LBD system (Mejia and Kajikawa), and the automatic construction of LBD knowledge infrastructure components (Škrlj et al.).

Future LBD systems must recognize the heterogeneity of gaps in the literature and be capable of evaluating the relative merits of bridging one gap to another. Not all disconnections in literature are created equal. The first paper by Peng et al. tackles this problem. The authors defined “gaps” as topics one should expect to occur together in the literature but are unexpectedly missing. The authors reported that gap-filling PubMed articles related to human diseases were cited more frequently than non-gap-filling ones and were more likely to be published in high-impact journals. This result is reassuring, validating the values of LBD as a useful knowledge discovery tool. The paper raised many interesting questions and far-reaching implications for LBD research. For example, would one be better off filling the easier “low-hanging fruit” gaps instead of those representing disciplinary communication gaps even though bridging the latter might lead to more transformative outcomes?

Usability studies must be a central theme of LBD research (Smalheiser et al., 2006; Smalheiser, 2012) but there is limited research output on this topic (Phang et al., 2022). The second paper in this collection demonstrates how the classic linking term count technique remains an effective LBD approach when combined with data obtained from high throughput metabolomic analyses (Henry et al.). Its output, validated *in vivo*, uncovered novel metabolomic pathways which suggested lecithin cholesterol acyltransferase as a druggable target for cardiac arrest. This article is the only known example of LBD application in metabolomics, indicating the field's expanding research frontiers. More importantly, Henry et al. work provided crucial insights into user interactions with an LBD tool and documented that the way LBD output is displayed to the users was critical to the LBD system's effectiveness. Surprisingly, users benefited from the system's displaying well-known direct connections between terms *in addition* to implicit logical ABC connections. The authors argued it is a crucial usability element because it gives users confidence that the system can detect meaningful information. This contrasts with the prevailing notion that LBD systems should only present previously unknown indirect connections between terms. Another remarkable observation is the users' ability to quickly skim past generic terms in the output, thus downplaying the importance of pre-eliminating generic terms as typically advocated by classical LBD approaches. This article leaves an important footprint in our quest to understand LBD human-computer interactions.

There is good progress in advancing the domain independence, scalability, and visual capabilities of LBD systems. The third paper by Mejia and Kajikawa showed us that literature-based discoveries could produce meaningful results in domains where controlled vocabularies do not exist, such as social sciences. Using food security and Internet-of-Things topics as an example, this article demonstrates that the semantic similarity of B terms could be modeled and measured based on the natural clustering in a large-scale network of interconnected terms, in the absence of any strong ontology resources. High-quality data visualizations helped users spot interesting implicit connections more easily. When combined with network and cluster analyses, shallow text mining techniques produced a highly scalable LBD approach that does not compromise its discovery power.

Knowledge-based infrastructures are needed that enable more effective hypothesis generation, preferably in an automated fashion. The final piece in this Research Topic is an excellent contribution that showcases the possibility of reconstructing a physical protein network solely from MeSH literature annotations with high accuracy. Škrlj et al. introduced CHEMMESHNET, a large literature-based network of millions of protein interactions extracted from chemical MeSH keywords in the PubMed database. Literature-based discovery tasks can then be performed on this network to find novel protein-protein interactions.

Literature-based discovery is a maturing field. Through this editorial, we hope to spark fresh ideas and rejuvenate research interests in diverse and emerging aspects of LBD. We trust that readers will find food for thought and enjoyment in our latest Research Topic collection.

## Author contributions

All authors listed have made a substantial, direct, and intellectual contribution to the work and approved it for publication.
